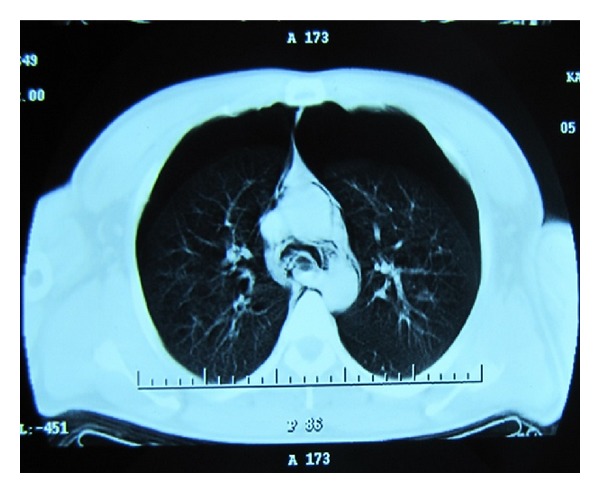# Bilateral Spontaneous Pneumothorax, Pneumomediastinum, and Subcutaneous Emphysema: Rare and Fatal Complications of Asthma

**DOI:** 10.1155/2012/242579

**Published:** 2012-12-23

**Authors:** Zeynep Karakaya, Şerafettin Demir, Sönmez Serkan Sagay, Olcay Karakaya, Serife Özdinç

**Affiliations:** ^1^Department of Emergency Medicine, Adana State Hospital, Adana, Turkey; ^2^Department of Cardiology, Adana State Hospital, Adana, Turkey; ^3^Department of Thoracic Surgery, Adana State Hospital, Adana, Turkey; ^4^Department of Radiology, Adana State Hospital, Adana, Turkey; ^5^Department of Emergency Medicine, Afyon Kocatepe University, Afyonkarahisar, Turkey

## Abstract

Simultaneous bilateral spontaneous pneumothorax (SBSP) and pneumomediastinum are complications rarely observed synchronously during an acute asthma attack. It is a clinical condition that manifests itself with serious respiratory distress and must be rapidly diagnosed and treated. Although bilateral spontaneous pneumothorax has already been reported in asthma patients in the literature, its concurrence with subcutaneous emphysema and pneumomediastinum is extremely rare except for iatrogenic conditions. By sharing this case about a 39-year-old patient who presented to the emergency room with severe respiratory distress and developed cardiopulmonary arrest during his physical examination, our aim is to emphasize that a rapid diagnosis and treatment by the emergency physicians is the only way for survival in these patients.

## 1. Introduction

 Asthma is a disease characterised by symptoms including wheezing, dyspnea, cough, tightness in the chest, chronic airway inflammation and increased airway resistance [1]. Simultaneous bilateral spontaneous pneumothorax (SBSP), subcutaneous emphysema, and pneumomediastinum that develop during an asthma attack are important and life-threatening complications that are observed singly or rarely synchronously. Collection of air in the pleural cavity and the subsequent collapse of the lungs is a condition known as pneumothorax [2]. The ratio of simultaneous bilateral spontaneous pneumothorax is approximately 1.3% among all cases of pneumothorax [3]. In patients with SBSP, the incidence of an underlying lung disease is greater than in the patients with unilateral spontaneous pneumothorax [4]. Pneumomediastinum is the presence of gas or free air in the mediastinum. Although it is generally a benign condition, its concurrence with pneumothorax may prove fatal during a serious asthma attack. A rapid diagnosis and treatment may be life-saving in these patients.

## 2. The Case

**Figure 1:**
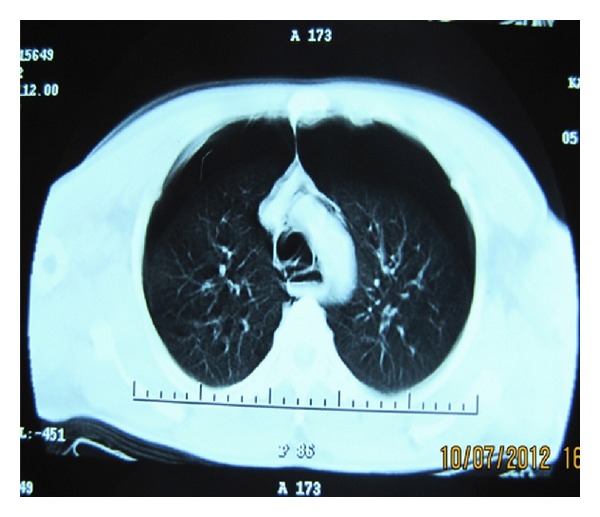


A 39-year-old male patient was brought to the emergency room with respiratory distress and chest pain. The patient's general condition was poor and his mental state was agitated. His BP was 100/60 mmHg; the heart rate was 130 bpm and his respiratory rate was 33 breaths per minute. The patient was cyanotic and the pulse oximeter revealed an oxygen saturation of 68%. Since the patient went into respiratory arrest and lost consciousness during the examination, a rapid endotracheal intubation was performed in order to keep the airway open and respiration was supported with the help of a bag valve mask. In the auscultation of the lungs, reduced respiratory sounds were observed in both hemithoraces and the crepitation pointed to subcutaneous emphysema in the neck and shoulders. The patient was connected to a mechanical ventilator and posteroanterior lung radiograph and thoracic CT were requested. The images revealed bilateral pneumothoraces of 4 cm on the right and 5 cm on the left sides. A pneumomediastinum was observed around the heart, together with subcutaneous emphysema (Figures 1 and 2).

Tube thoracostomy was applied to both lungs using 28 F thoracic drains through the 4th intercostal space on the midaxillary line. Air leaks were observed in both thoraces. No tracheal injury was present. The patient was connected to a mechanical ventilator to be followed up. The patient history revealed that he was already diagnosed with asthma and was receiving irregular treatment. In the intensive care unit, the patient was administered salbutamol against the asthma. He was extubated during his second day of hospitalisation and was taken to the ward on the third day. Also on the third day, chemical pleurodesis with talcum powder was performed on his right lung, followed by his left lung on the next day. When pulmonary expansion was observed during the followup of the patient, his thoracic tubes were removed and he was discharged.

## 3. Discussion

 Presence of air in the pleural cavity without a history of trauma is defined as spontaneous pneumothorax. If the condition occurs without an underlying disease, it is described as a primary spontaneous pneumothorax, whereas it is called a secondary spontaneous pneumothorax if it is brought about by a concurrent disease [2]. Unlike in unilateral spontaneous pneumothorax, the incidence of an underlying pulmonary disease is higher in patients with SBSP and the condition is most frequently related to Chronic Obstructive Pulmonary Disease [4]. There are studies showing that this complication is more frequent in males [5]. Patients with SBSP may present with various clinical symptoms from a mild dyspnea to cardiopulmonary failure [5]. Although the chest X-ray is a standard procedure, thoracic tomography gives superior results in terms of small pneumothoraces and pneumomediastinum. The aim of the treatment is to achieve pulmonary expansion and to prevent a recurrence. The tube thoracostomy is a method applied during the acute phase. Also in our case, the patient was applied a tube thoracostomy and chemical pleurodesis was performed using talcum powder. While chemical pleurodesis may control difficult or recurrent pneumothoraces, since surgical treatment modalities are more efficient, it is only applied when the patient rejects surgical intervention or when no surgical procedure can be performed on the patient [6]. Since our patient rejected a surgical approach, a chemical pleurodesis was performed. 

Pneumomediastinum is described as the presence of gas or free air within the mediastinum. Despite the various causes which lead to this problem, it is a rarely observed clinical condition. Although pneumomediastinum is most frequently associated with asthma attacks, it may also occur due to barotraumas, intrathoracic pressure increases, the Valsalva manoeuvre, and withdrawal symptoms of cocaine or similar substances. In our patient, the pneumomediastinum had developed due to a serious asthma attack. In severe acute asthma, pneumomediastinum develops because of the overexpansion of the distal air ways due to the obstruction in the minor air ways and the subsequent alveolar rupture. Because of the pressure difference, the air in the pulmonary interstitium moves in the centripetal direction from the pulmonary parenchyma towards the mediastinum [7]. This movement explains the occurrence of pneumomediastinum. Its classical symptoms include chest pain, difficulty in swallowing, and dyspnea varying according to the degree of the compression. The diagnosis is made clinically and radiologically following the suspicion by the clinician. The typical finding is crunching and rasping sounds synchronous with the peak of the heart beat during the auscultation on the front side of the chest (Hamman's sign) [8]. The air may pass below the skin and may flow towards the neck and the face, leading to subcutaneous emphysema [9]. In our patient, subcutaneous emphysema was especially manifest in the neck. Although pneumomediastinum is generally a benign and self-limiting condition that responds to conservative therapy, serious complications such as high blood pressure and/or bilateral pneumothorax, as well as cardiac compression and high pressure causing a reduction in the cardiac output, have been reported in pneumomediastinum [10]. In our case, cardiopulmonary arrest developed right after the presentation of the patient. Although the concurrence of bilateral pneumothorax, pneumomediastinum, subcutaneous emphysema, and asthma is rarely observed, it may obviously progress very fatally. Thus, in case of chest pain and dyspnea, this condition may also be considered in the differential diagnosis. 

In conclusion, SBSP is a clinical condition that is treated according to the age, clinical status, and the underlying causes. Although it is usually benign in young patients, it may become life-threatening in advanced ages and in patients with limited pulmonary reserve, unless it is urgently managed. Misdiagnosis and delayed treatment can lead to tension pneumothorax, and the patient's death [11]. The clinician's suspicion is the most important starting point in the diagnosis, and a timely diagnosis and the appropriate treatment may prevent mortality and morbidity. 

## Figures and Tables

**Figure 2:**